# Role of CEACAM1 and CEACAM20 in an In Vitro Model of Prostate Morphogenesis

**DOI:** 10.1371/journal.pone.0053359

**Published:** 2013-01-24

**Authors:** Hui Zhang, Andreas Eisenried, Wolfgang Zimmermann, John E. Shively

**Affiliations:** 1 City of Hope Irell & Manella Graduate School of Biological Sciences, Duarte, California, United States of America; 2 Department of Immunology, Beckman Research Institute of City of Hope, Duarte, California, United States of America; 3 Anästhesiologische Klinik, Universitätsklinikum Erlangen, Erlangen, Germany; 4 Labor fürTumorimmunologie, LIFE-Zentrum, München, Germany; The Chinese University of Hong Kong, Hong Kong

## Abstract

CEACAM20, a novel member of the CEACAM1 gene family with expression limited to the lumen of small intestine, testes, and prostate, is co-expressed with CEACAM1 in adult prostate tissue and down-regulated to the same extent as CEACAM1 in prostate cancer. Since prostate cancer often involves loss of epithelial lumen formation, we hypothesized that CEACAM20 and CEACAM1 play important roles in lumen formation of normal prostate epithelium. When prostate cells were grown on Matrigel as a source of extracellular matrix (ECM), they differentiated into acinar structures with single tubules and well-defined lumina closely resembling embryonic prostate organoids. Confocal microscopic analysis revealed restriction of CEACAM20 to acini and CEACAM1 to tubule structures, respectively. Inhibition of CEACAM1 with antibodies or soluble CEACAM1 or antisense oligonucleotides inhibited tubule formation by over 50% while the remaining tubules were stunted. Inhibition of CEACAM20 with antisense oligonucleotides completely inhibited tubule formation and stunted the growth of acini. We conclude that CEACAM20 and CEACAM1 not only mark the lumina of adult prostate tissue but also play a critical role in the vitro generation of prostate organoids.

## Introduction

The carcinoembryonic antigen-related cell adhesion molecule (CEACAM) gene family, a subgroup of the immunoglobulin superfamily, has 12 genes located on human chromosome 19. Their gene products mediate cell-cell adhesion among multiple cell types including epithelium, endothelium and lymphocytes, regulating diverse signal pathways including vasculogenesis [1], insulin clearance [2], cell growth [3] and apoptosis [4]. Recently, nine new members of the CEACAM gene family were discovered [5], among which CEACAM20 is unique with a truncated IgV-like N domain (38 amino acids compared to 108–110 amino acids for other CEACAM family members). In addition, its unusually long cytoplasmic domain has an immunoreceptor tyrosine-based activation motif (ITAM) conserved across the mouse, rat and human CEACAM20 genes. CEACAM20 transcripts are restricted to the reproductive system (prostate and Leydig cells) and the intestinal tract (colon, jejunum, ileum and cecum) [5]; however its function has not been studied. Since prostate cancer is the second leading cause of cancer deaths in men (CDC report in 2007), we began functional studies of CEACAM20 in the prostate.

The high life-time incidence (1 out of 6) and mortality (1 out of 32) of prostate cancer make it an important health problem. Although early diagnosis followed by early treatment increases survival, no or few symptoms occur at an early stage for prostate cancer. The Gleason grade of prostate cancer exhibits the best correlation with degree of malignancy, prognosis and treatment. The gradual loss of glandular lumina is the major change in the transition from low (1–3) to high (4–5) Gleason grade. CEACAM1 is highly expressed on the surface of normal prostatic luminal glands, whereas its loss of expression occurs in high Gleason grade cancer sections that lack lumina [6]. We found a similar expression pattern for CEACAM20 in normal vs malignant prostate using immunohistochemistry staining (IHC), suggesting the possibility that down regulation of CEACAM1 and/or CEACAM20 is responsible for the absence of lumina in high Gleason grade prostate cancer. Previous studies on CEACAM1 in prostate cancer show that on the one hand, CEACAM1 inhibits prostate cancer growth [7], and on the other hand, CEACAM1 is upregulated on endothelial cells of blood vessels in prostate tumors [8].

In support of a role for CEACAM1 in regulation of lumen formation in the mammary gland, an organ that shares structural similarity with normal and malignant prostate, we have found that mammary epithelial cells lacking CEACAM1 fail to form lumina in Matrigel culture while forced expression of CEACAM1 in breast cancer cells restores lumen formation [4]. Based on the above function of CEACAM1 in mammary epithelial cells, we hypothesized that CEACAM1 and CEACAM20 would play a role in the maintenance of the normal phenotype of prostate epithelial cells. We found high expression of CEACAM20 in frozen tissue sections of human prostate as well as in normal human prostate epithelial cells (hPrECs), at both the mRNA and protein levels. Using Matrigel culture (on-Matrigel) as a model for epithelial cell differentiation, we observed that hPrECs formed organoids firmly attached to extracellular matrix (ECM) with a tubule extending out from the acini of organoids into the fluid media. CEACAM1 was exclusively expressed on the tubules while CEACAM20 was exclusively expressed on the spherical acini and in the lumen. Blocking CEACAM1 with soluble CEACAM1 or anti-CEACAM1 antibody significantly blocked tubule formation in a CEACAM1-specific, dosage and temporal dependent manner. In addition, knocking down CEACAM1 or CEACAM20 with antisense oligonucleotides using a gymnotic delivery method [9] blocked hPrECs differentiation into organoids. These studies suggest that CEACAM1 and CEACAM20 play a significant role in normal prostate epithelial cell differentiation.

## Materials and Methods

### Antibodies and antigens

The following antibodies were used: CEACAM20 mAb (6G4A5, Aldevron), cytokeratin 8/18 mAb and Alexa 488, Alexa 555 or Alexa 647-conjugated goat anti-mouse IgG, Alexa 594 conjugated phalloidin (Life Technologies), PAP mAb (PASE/4LJ), high molecular weight cytokeratin mAb (34βE12), prostate specific antigen (PSA) mAbs (Dako), PE-CD133 mAb (AC133 and 293C3, Miltenyi Biotec), APC-CD56 mAb (eBioscience), β-actin and androgen receptor (AR) mAbs (Santa Cruz). CD33 mAb was a kind gift from Dr. David Colcher (City of Hope) and used as an isotype control. CEACAM5 mAb (T84.66 and T84.1) [1], [10] and soluble-CEACAM1 [11] was generated in house. CEACAM1 mAb (5F4) was from Dr. Richard Blumberg (Harvard University).

### Cell Culture

Normal human prostate epithelial cells (hPrECs), isolated from a healthy donor, were purchased from Lifeline Cell Technology (Frederick, MD) and cultured with ProstaLife Medium Complete Kit, serum-free ProstaLife Basal Medium supplemented with 6 mM L-glutamine, 0.4% Extract PTM, 1 µM Epinephrine, 0.5 ng/mL rh TGF-α, 100 ng/mL hydrocortisone hemisuccinate, 5 µg/mL rh insulin, 5 µg/mL apo-transferrin and 1% Antibiotic-Antimycotic (Gibco). HEK293 cells were got from ATCC (Manassas, VA). Human peripheral blood mononuclear cells (PBMC) were isolated as previously reported [12].

### RNA Extraction, PCR and quantitative PCR

The use of frozen discarded anonymous tissue sections without the need for informed consent were obtained from the Pathology Core Lab of City of Hope approved by the City of Hope Institutional Review Board, IRB number 03020. 20 µm-thin frozen prostate tissue sections were homogenized and lysed in buffer RLT plus (from RNAeas kit, Qiagen) or RIPA (Sigma) supplemented with Benzonase (v∶v, 1∶2000, Novagen). Total mRNA was extracted with RNAeasy kit or purchased from Cells Applications Inc (San Diego, CA). PCR and Q-PCR were performed as previously reported [13]. The primers for PCR and Q-PCR are listed in **Table S1A** and **Table S1B**, respectively.

### CEACAM20 cloning

Total RNA was isolated from cryosections (20–50 µm) from human small intestine and converted to cDNA using a RT-PCR kit and random hexamer oligonucleotides as primers (Promega, Madison, USA). CEACAM20 cDNA was amplified by PCR using the High Fidelity Enzyme Mix (Fermentas, St. Leon-Rot, Germany) and *Hin*dIII and *Eco*RI-containing primers binding to the 5′- and 3′-untranslated regions, respectively (**Table S1C**). After digestion with *Hin*dIII and *Eco*RI CEACAM20 was cloned into the pcDNA3.0 vector (Invitrogen).

HEK293 cells were stably transfected with pcDNA3.0-CEACAM20-5L using electroporation [14] and selected by FACS. MCF7 cells transfected with CEACAM1 were previously described [4].

### Western blotting, Hematoxylin and eosin stain (HE stain), Immunohistochemistry staining (IHC) and FACS

Cells were lysed as previously reported [15]. 5F4 or 6G4A5 (2 µg/mL) were used for flow analysis. HE and IHC staining of prostate tissue and cells on Matrigel was performed as previously reported [4], [16] using 6G4A5 (40 µg/ml) or 5F4 (20 µg/mL). Surface and intracellular staining were performed as previously published [12]. Cell sorting was performed using a FACSAria I (BD Biosciences).

### Matrigel culture and inhibition assay

Phenol red free Matrigel (BD) was used to avoid an estrogenic effect [17]. hPrECs (1.0×10^5^) were added to solidified Matrigel and after 4 to 6 h, unattached cells were removed and 2 mL of fresh medium was added. Since cells were plated on Matrigel, the system is referred as on-Matrigel. In addition, cells were plated in Matrigel as previously published [16]. For inhibition assays, soluble-CEACAM1 or antibodies (T84.1, T84.66 or isotype control) were added in medium every three days. Colonies with or without tubule formation were counted with an inverted light microscope.

### hPrECs recovery from Matrigel

Cells on Matrigel were washed with PBS once before incubation with 1 mg/mL dispase solution (Invitrogen, dispase was dissolved in ProstaLife Medium Complete Kit) for 1 h at 37°C [18]. Cells and melted gel were collected with 15 mL Falcon tube (BD biosciences) and centrifuged at 800 g for 5 min. Supernatants were removed. Cell pellet was resuspended in 2 ml of 0.05% (wt/vol) Trypsin/EDTA (Gibco) and incubated at 37°C for 5 min to dissociate the cells in the same acinus. Trypsinized cells were washed with PBS and spinned down for the further FACS, RNA extraction or western blot experiments.

### Confocal and electron microscopy (EM)

For confocal microscopy, hPrECs were fixed, permeabilized and stained as previously reported [13]. Cells were incubated with 2 µg/mL anti-CEACAM1 (5F4) or anti-CEACAM20 (6G4A5) for 24 h at 4°C. For EM, cells were treated as previously described [19]. EM was performed on an FEI Tecnai 12 transmission electron microscope equipped with a Gatan Ultrascan 2K CCD camera. For nanogold immunostaining experiments, samples were frozen in the Leica EM PACT-2 high pressure freezing unit. Substitution was frozen in 0.4% GA +0.1%UA and lowicryl resin was embeded in Leica EM AFS2 followed by blocking in 10% Normal Donkey Serum in TBS with 0.1% Tween, pH 7.6, for 15 minutes. Sections on the grid were incubated with 40 µg/mL anti-hCEACAM1 mAb (5F4) or anti-hCEACAM20 (6G4A5) primary antibody followed by colloidal gold-conjugated secondary antibody.

### Time lapse microscopy

Twelve hours after hPrECs were attached on Matrigel, plates were transferred to a Weatherstation precision control stage incubator on an Olympus 1X2-UCB inverted fluorescent microscope equipped with an Orca-ER Hammamatsu camera. Phase contrast images were taken every 30 min. Movies were processed with Final Cut Pro (Apple, Inc).

### Gymnotic delivery of antisense oligonucleotides

Phosphorothioate based 16mer antisense oligonucleotides to CEACAM1, CEACAM20 and a scrambled control (**Table S1D**) that increased RNase resistance by incorporation of 2′-O-(2-methoxyethyl) (2′-O-MOE) and 2′-fluro-ribose sugar modifications [20] were synthesized by the Nucleic Acid/Protein Service Core (City of Hope). hPrECs (passage 4) were split at a ratio of 1∶16 one day before adding antisense oligonucleotides (0.25–1.0 µM). Fresh 16mer antisense oligonucleotides were added every three days when medium was changed. Cells reached 90% confluence 10 days after treating with 16mer antisense oligonucleotides and were transferred on Matrigel. Regular medium was changed every three days thereafter.

### Statistical Analysis

All assay data are expressed as means ± standard deviation. Unpaired Student's *t*-tests were used for comparisons. All P values were two sided and calculated with GraphPad Prism software (version 5.0, GraphPad Software, San Diego, CA, USA).

## Results

### CEACAM20 expression in human prostate tissue

**Figure 1:**
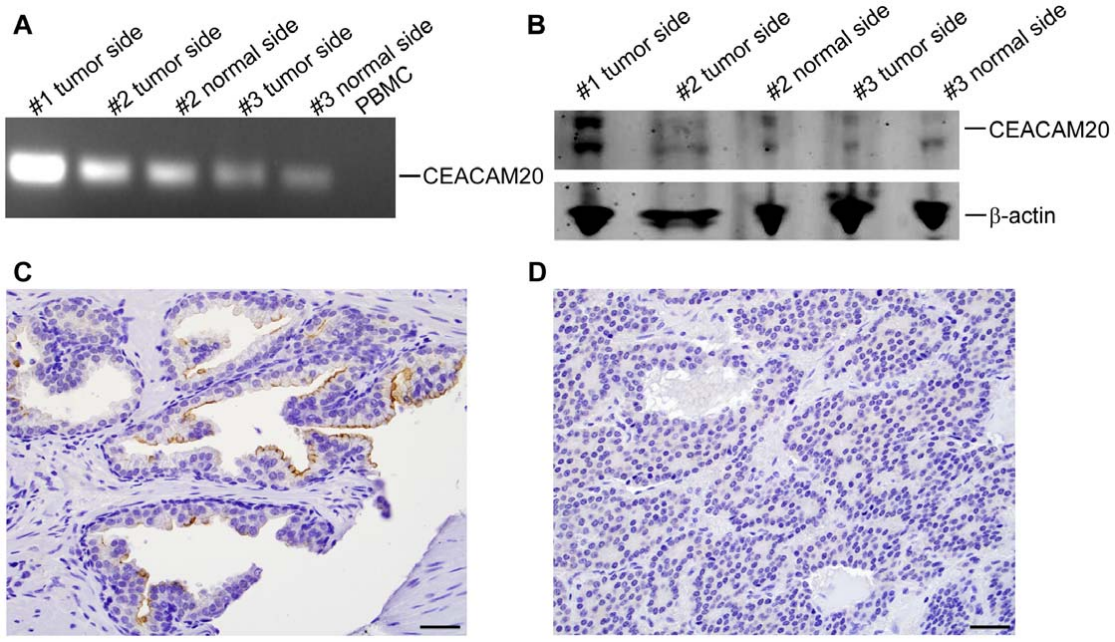
CEACAM20 expression in normal and malignant prostate. **A**. RT-PCR analysis for CEACAM20 of three prostate tumors including normal and malignant tissue with PBMCs as a control. **B**. Western blot analysis of the same tissues with anti-CEACAM20 monoclonal antibody 6G4A5 with anti- β-actin as a control. Immunohistochemistry staining of normal (**C**) and malignant (**D**) prostate with anti-CEACAM20 mAb, scale bar: 20 µm.

RT-PCR analysis of CEACAM20 expression in different human tissue RNAs showed that CEACAM20 was mainly expressed in the small intestine and prostate (**Fig. S1**). Given the interest in the identification of new markers for prostate cancer, we isolated mRNA and proteins from frozen prostate tissues and found that both CEACAM20 mRNA and protein was expressed in normal prostate and prostate tumors (**Fig. 1A and 1B**). IHC staining of normal and prostate tumor sections revealed that CEACAM20 was expressed on the luminal surface of prostate glands (21/22 of normal prostate and 17/17 of prostate tumors with Gleason grade ≤3) but its expression was absent in prostate tumors with a Gleason grade ≥4 (6/6) lacking lumina (**Fig. 1C** **and** **Fig. 1D**). In this respect, the CEACAM20 IHC staining pattern resembled CEACAM1 expression in the normal and malignant prostate [6].

The finding that CEACAM1 and CEACAM20 staining was confined to the lumina in normal glands and the absence of staining in malignant glands lacking lumina, suggested the possibility that down-regulation of both CEACAM1 and CEACAM20 was responsible for the absence of lumina in prostate cancer. Our previous work on breast cancer cells has shown that CEACAM1 is essential to maintain normal lumenal structure in-Matrigel culture [4], [16], [21], culture conditions in which cells are completely surrounded by solid Matrigel. Since CEACAM20 exhibits an expression pattern in prostate similar to CEACAM1, we explored their function in the differentiation of the prostatic lumen in-Matrigel culture. In order to perform these studies, we selected primary prostate epithelial cells (hPrEC) which were first subjected to phenotypic analysis.

### Phenotypic analysis of primary human normal prostate epithelial cells on plastic

**Figure 2:**
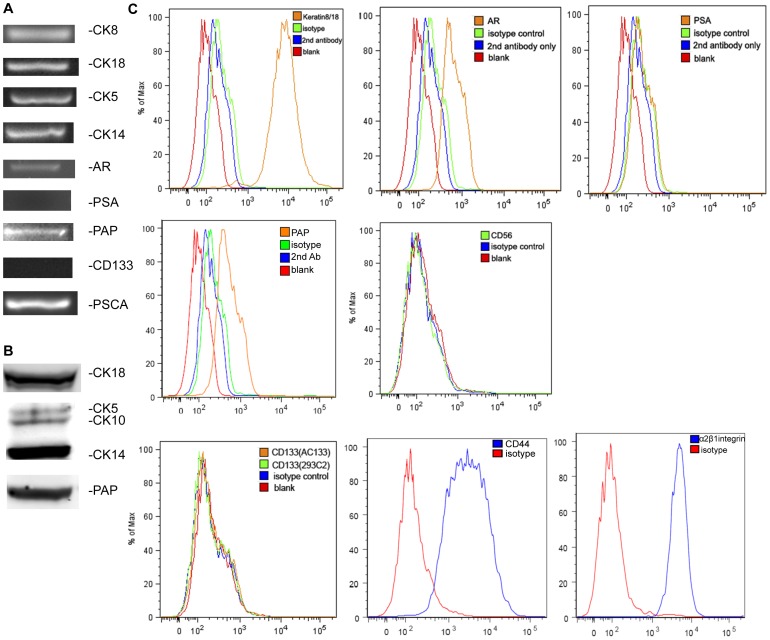
Phenotype analysis of hPrECs grown on plastic. (**A**) PCR analysis for CK8, CK18, CK5, CK14, AR, PSA, PAP, CD133 and PSCA. (**B**) Western blot analysis for CK18, CK5, CK14 and PAP. (**C**) FACS analysis for CK8/18, AR, PSA, PAP, CD56, CD133, CD44 and α_2_β_1_ integrin.

To explore the possible role of CEACAM1 and CEACAM20 in prostatic lumen formation, we phenotyped the primary prostatic cell line hPrEC grown on plastic. Since prostate epithelium is composed of luminal, basal and neuroendocrine cells, it was necessary to determine if all three type of cells were present. hPrECs were positive for both luminal markers, cytokeratin 8/18, and basal makers, cytokeratin 5/14 (**Fig. 2A, 2B and 2C**) but negative for neuroendocrine marker CD56 (**Fig. 2A and 2C**). The cells also expressed androgen receptor (AR) and prostate acid phosphatase (PAP) but not prostate specific antigen (PSA) at both the mRNA and protein level (**Fig. 2A, 2B and 2C**). Since only intermediate cells, located between basal and luminal cells, express both CK8/18 and CK5/14, we performed further analysis. Previous work demonstrated that CD133^−^ α2β1 integrin^+^ CD44^+^ cells are considered as the transit amplifying population or intermediate cells [22]. As shown in **Figure 2A and 2C**, hPrECs were negative for CD133, but positive for CD44 and α2β1 integrin. hPrECs were also positive for prostate stem cell antigen (PSCA; **Fig. 2A**), a marker of intermediate prostate epithelia cell and not expressed on the basal cells. Based on the above findings, we conclude that hPrECs are intermediate prostate epithelial cells.

**Figure 3:**
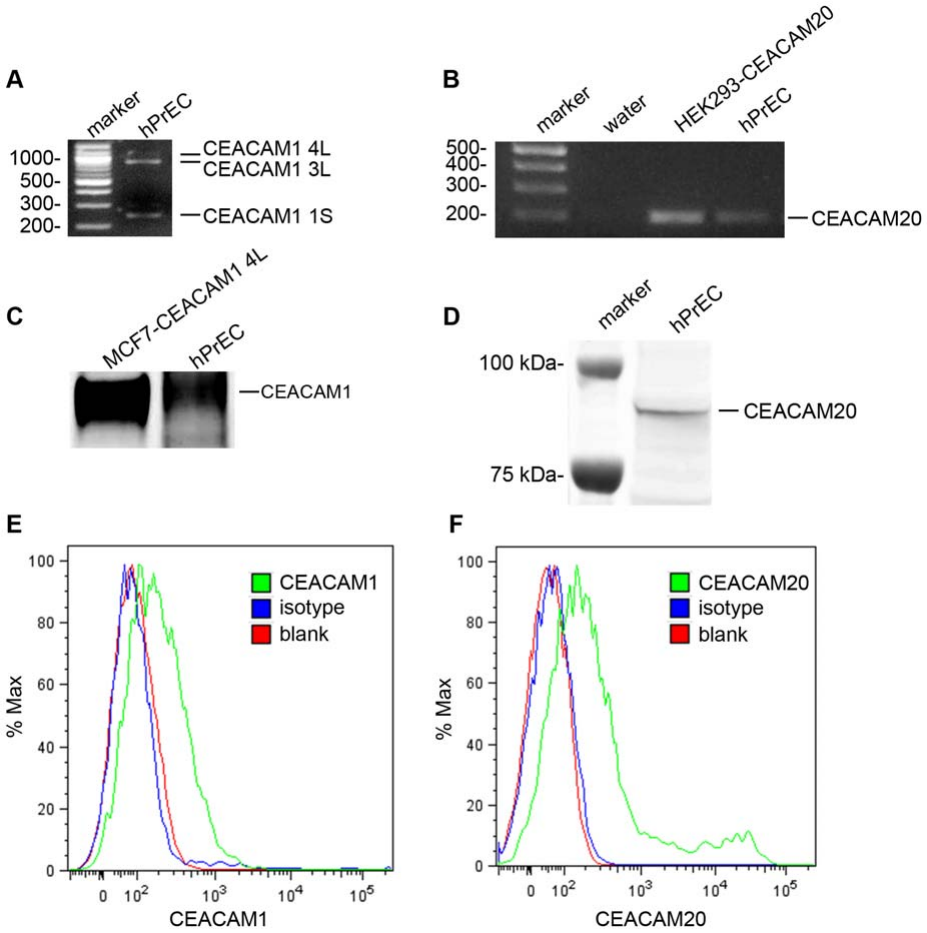
CEACAM1 and CEACAM20 expression hPrECs. Detection of CEACAM1 (**A**) and CEACAM20 in hPrEC and CEACAM20 transfected HEK293 cells (**B**) by RT-PCR. Western Blot analysis of CEACAM1 in CEACAM1 transfected MCF7 mammary cells and in hPrEC cells (**C**). Western blot analysis of hPrEC cells (**D**). Detection of CEACAM1 (**E**) and CEACAM20) in hPrEC cells by flow cytometry analysis, mouse-anti-human IgG1 was used as the isotype control.

Since normal prostatic epithelium also expresses CEACAM1 and CEACAM20, the focus of our study, we also analyzed these cells for these two cell surface markers. CEACAM1 and CEACAM20 expression was detected at the mRNA level (**Fig. 3A and 3B**) and the protein level by both Western Blot (**Fig. 3C and 3D**) and flow analysis (**Fig. 3E and 3F**).

### hPrECs form acini in-Matrigel and organoids on-Matrigel

**Figure 4:**
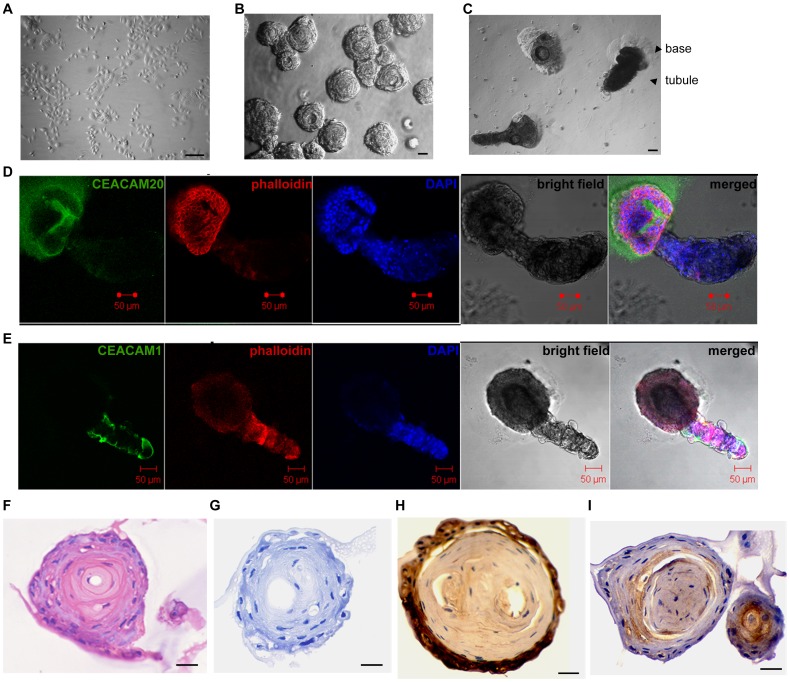
Formation of organoids by hPrEC cells in-Matrigel or on-Matrigel. Morphology of hPrECs grown on plastic (**A**), **in-Matrigel** (**B**), and **on-Matrigel** (**C**), scale bar 50 µm. Confocal microscopy analysis of organoids on-Matrigel stained with anti-CEACAM20 (green) (**D**) or anti-CEACAM1 (**E**) (green), phalloidin (red) and DAPI (blue), scale bar 50 µm. HE staining (**F**) and immunohistochemisctry staining of isotype control (**G**), CEACAM20 (**H**) and CEACAM1 (**I**), scale bar 20 µm.

The in-Matrigel culture system developed by Bissell and coworkers [23] simulates the in vivo environment cells encounter better than the traditional plastic culture system, and also permits cell differentiation [24]. Since the normal mammary epithelial cell line MCF10F expresses CEACAM1, and can form lumina in-Matrigel culture [4], [16], we predicted that hPrECs would also form lumina in-Matrigel. Indeed, when hPrECs were cultured in-Matrigel, the cell morphology changed dramatically from spindle-like (**Figure 4A**) to spherical acini (**Fig. 4B**). In addition, hPrECs were cultured on the surface of Matrigel (on-Matrigel), a culture system that also is known to induce cell differentiation [25]. In this culture system, hPrECs form organoids firmly attached to Matrigel with a tubule extending out from the organoids into the fluid media (**Figure 4C**). In order to further observe the formation of these organoids, we performed time-lapse photography to follow hPrECs migration and differentiation from days 1 to 7 (**Movie S1**). By day 1, hPrECs formed web-like structures in which individual colonies had long, extended epithelial cords that reached out to nearby colonies. By day 2 colonies merged and formed spherical acini. By day 3 buds appeared on the surface of acini and by day 4 the buds developed into mature tubule-like structures. Compared to in-Matrigel culture, on-Matrigel culture organoids closely resemble the acinar-budding-branching morphogenesis characteristic of prostate development [26], [27]. This is noteworthy, in that Witte and co-workers [28] proposed that basal cells are stem cells with the capacity to differentiate, but we now show that even intermediate epithelial cells can differentiate when cultured on-Matrigel.

Although the role of several signal pathways has been studied in prostate branching morphogenesis, the potential roles of CEACAM1 and CEACAM20 have not been studied. Using confocal microcopy, we found that CEACAM1 was exclusively expressed on the surface and interior of tubules, while CEACAM20 was expressed on the surface and interior of lumena (**Fig. 4D and 4E**). In addition, we observed membranous secretion of CEACAM20 into the surrounding Matrigel (**Fig. 4D**), which suggests that CEACAM20 can be secreted. To further confirm the expression pattern of CEACAM1 and CEACAM20 in the organoids, organoids were fixed, embedded and H&E and immunohistochemistry staining was performed. H&E staining (**Fig. 4F**) shows a clearly defined lumen, as well as in the immunostaining control (**Fig. 4G**). CEACAM20 immunostaining was restricted to the exterior cells of acini (**Fig. 4H**) and CEACAM1 staining to interior cells (**Fig. 4I**). Lack of detectable CEACAM1 or CEACAM20 in the lumen might be due to the washing step involved in the H&E immunostaining (**Fig. 4H–I**) but not an issue in the confocal staining (**Fig. 4D**). Since secretion is the characteristic function of the prostate and CEACAM1 is secreted into multiple human biological fluids [19], [29], [30], TEM and nanogold immunostaining were performed. Abundant vesicles, associated with active secretion, were observed in the lumen and beneath the cell membrane for both acini and tubules (**Figure S2A and S2B**). Both CEACAM1 and CEACAM20 were found in the lumen by nanogold immunostaining (**Figure S2C and S2D**), indicating that differentiated hPrECs not only express CEACAM1 and CEACAM20 but also secrete these two molecules. Based on these results, we conclude (a) that spherical acini form first, followed by tubule formation, (b) both the spherical acini and tubules have central lumina, and (c) CEACAM1 and CEACAM20 have differential expression patterns, one restricted to tubules, the other to spherical acini but both can be secreted.

### Phenotypic analysis of primary human normal prostate epithelial cells on- Matrigel

**Figure 5:**
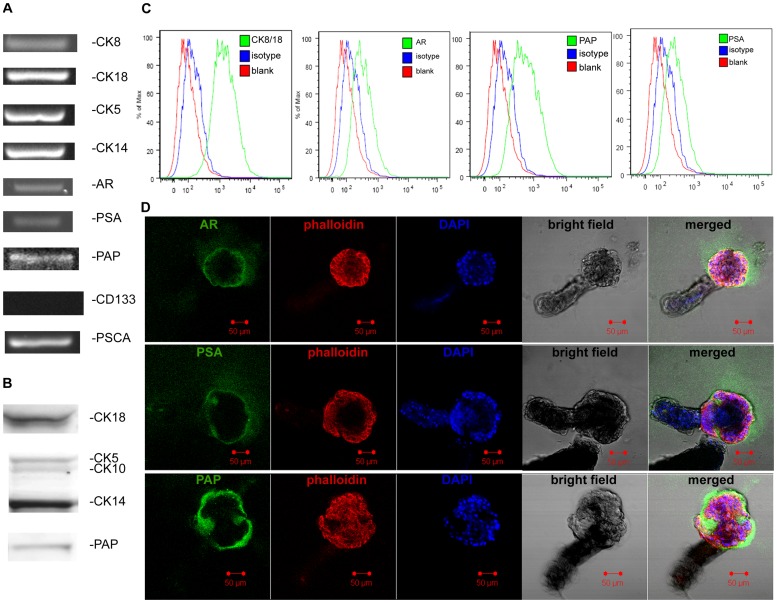
Phenotype analysis of hPrECs grown on-Matrigel. (**A**) PCR analysis for CK8, CK18, CK5, CK14, AR, PSA, PAP, CD133 and PSCA. (**B**) Western blot analysis for CK18, CK5, CK14 and PAP. (**C**) FACS analysis for CK8/18, AR, PAP and PSA, (**D**) Confocal microscopy analysis of organoids on-Matrigel stained with anti-AR (green) or anti-PSA (green) or anti-PAP (green), phalloidin (red) and DAPI (blue), scale bar 50 µm.

Since the hPrECs formed organoids on-Matrigel, it was necessary to perform additional phenotypic analysis. Essentially all of the intermediate cell markers were positive (**Fig. 5**). Thus, the intermediate markers were the same on cells grown on plastic or on-Matrigel. In addition, these cells now were positive for PSA (**Fig. 5A, 5C, 5D**). This indicates that these cells indeed have the ability to express PSA, but only on-Matrigel. Note that the pattern of secretion for PSA and PAP were similar to that for CEACAM20 by confocal analysis (**Fig. 5D**).

### Inhibition of tubule formation by anti-CEACAM1 antibody or soluble CEACAM1

To explore the function of CEACAM1 in hPrEC tubule formation, anti-CEACAM1 antibodies or soluble CEACAM1 (sCEACAM1) were added to the culture medium 6 hours after cells were plated on Matrigel, a time at which cells were firmly attached to the Matrigel. We have previously shown that anti-CEACAM1 mAb T84.1 or sCEACAM1, a recombinant protein including the N, A1, B1 and A2 domains of CEACAM1, can successfully inhibit acinus formation of MCF10F cells cultured in- Matrigel [16]. It is likely that both treatments inhibit CEACAM1 by interaction with the N-domain of CEACAM1, which is responsible for its cell adhesion function [31], [32]. An anti-CEACAM5 mAb and a mouse IgG1 isotype mAb (CD33) were used as controls for the anti-CEACAM1 treatments. By day 5, 79% hPrECs formed well-differentiated acinar-tubule structures when untreated or treated with anti-CEACAM5 or isotype control antibodies (**Fig. S3A** and **S3B**). In contrast, cells treated with anti-CEACAM1 mAb or sCEACAM1 formed significantly less tubules (p<0.0001, **Figure S3A** and **S3B**). In addition, the specific mAb treatments exhibited a dose response from 10 µg/mL (46% tubule formation) to 20 µg/ml (38% tubule formation) to 50 µg/mL (33% tubule formation). Similar tubule inhibition and dose-dependent effect were seen in sCEACAM1 treatment (**Fig. S3A** and **S3B**).

**Figure 6:**
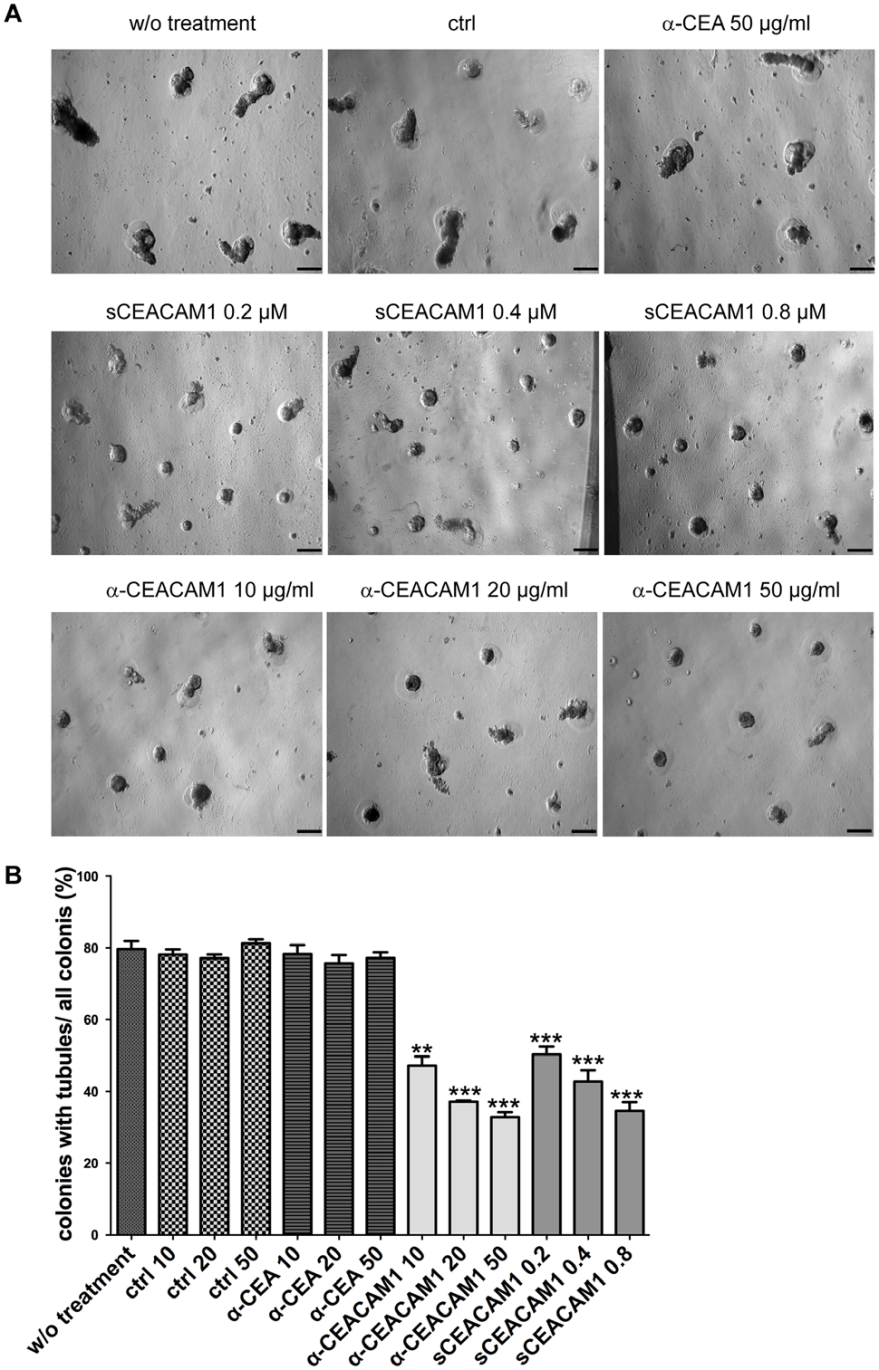
Inhibition of tubule formation by anti-CEACAM1 antibody or soluble CEACAM1. **A**. Morphology of untreated hPrECs grown on-Matrigel (CTRL), isotype control antibody treated, anti-CEA antibody treated (as a further control), anti-CEACAM1 antibody treated at 10, 20 and 50 µg/mL, and sCEACAM1 treated at 0.2, 0.4 and 0.8 µM for 11 days, scale bar 100 µm. **B**. Quantification of organoids with tubules (200 colonies counted for each treatment at day 11).

To test the possibility that anti-CEACAM1 or sCEACAM1 treatments might delay rather than inhibit tubule formation, we continued observing growth and differentiation of hPrECs for 11 days. As shown in **Figure 6**, the anti-CEACAM1 or sCEACAM1 treated groups had significantly less tubule formation compared with untreated or antibody control treated groups. Thus, we conclude that anti-CEACAM1 or sCEACAM1 treatments inhibit tubule formation in a CEACAM1-specific and dosage-dependent manner.

### Prostate tubule morphogenesis requires not only expression of the correct molecules but also their correct temporal expression

Since we observed the formation of tubule buds at day 3 followed by well-differentiated tubules at day 4, we divided the Matrigel culture time into two stages: stage one (days 0–2) in which no tubules were observed, and stage two (day 3 and after), the tubule forming stage. To determine the temporal pattern of CEACAM1's function, we added anti-CEACAM1 mAb or sCEACAM1 at day 2 and then counted the numbers of acini with or without tubules at days 5 and 11. Since no significant tubule inhibition was seen for either treatment when the start of treatment was delayed to day 2 (**Table S2**), we conclude that CEACAM1 expression/signaling was required for tubule formation as early as stage one before tubule buds were visually observable, but was dispensable at stage two when tubules had already formed. In addition, we attempted to inhibit tubule formation with anti-CEACAM20 mAb 6G4A5, but no significant tubule inhibition was observed (data not shown). However, the lack of activity of an anti-CEACAM20 antibody in a functional assay, does not rule out a possible functional role for CEACAM20 in tubule formation.

### Knock-down of CEACAM1 or CEACAM20 blocks differentiation of hPrECs on Matrigel

**Figure 7:**
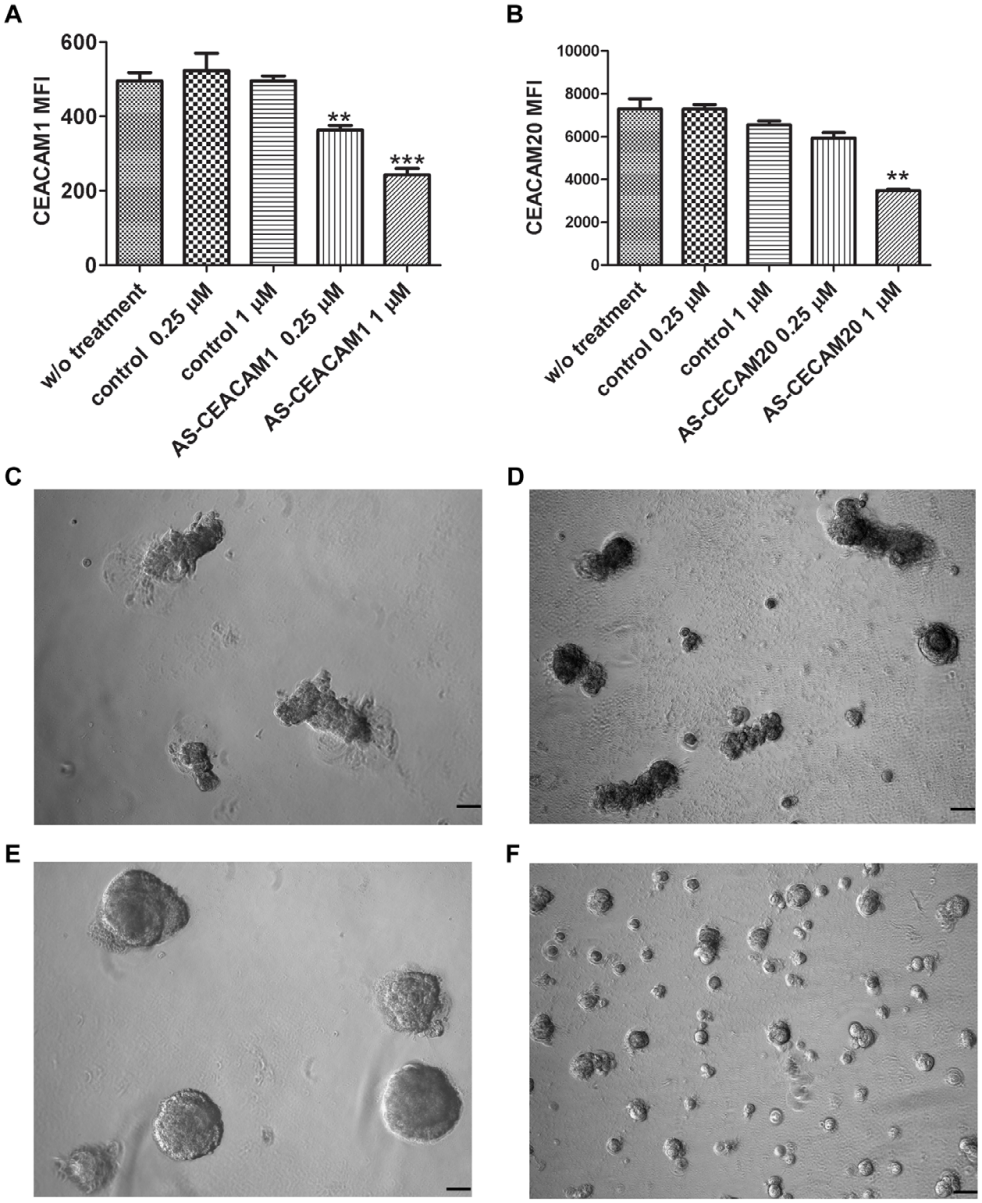
Inhibition of tubule formation with antisense oligonucleotides to CEACAM1 or CEACAM20. Efficiency of knockdown of CEACAM1 (**A**) or CEACAM20 (**B**) with antisense oligonucleotides administered by gymnosis and quantitated by measuring mean fluorescence intensity (MFI). Morphology of organoids after no treatment (**C**) or treatment with scramble control antisense (**D**) or antisense to CEACAM1 (**E**) or antisense to CEACAM20 (**F**), scale bar 100 µm.

To further study the function of CEACAM1 and CEACAM20 in hPrECs during Matrigel-induced morphogenesis, we attempted to transfect these cells with transfectamine mediated RNAi, but high toxicity of the transfection reagent killed hPrECs. We tested a new method termed “gymnosis” pioneered by Stein et al [9], [33]. In this method, cells take up nuclease resistant phosphorothioate locked nucleic acid based antisense oligonucleotides without the need of any delivery system but this method requires longer time periods and higher concentrations of antisense oligonucleotides to achieve target knock-down. Based on this idea, we synthesized phosphorothioates-based [34] antisense oligonucleotides with increased RNase resistance [35] by incorporation of 2′-O-(2-methoxyethyl) and 2′-fluoro ribose sugar modifications [20]. After 10 days of treatment with 1 µM CEACAM1 antisense 16 mer oligonucleotides, CEACAM1 surface expression was knocked down about 50% (**Fig. 7A**) and by western blot analysis (**Fig. S4C**) whereas mRNA transcript levels decreased by 30% (**Fig. S4A**). Cells formed acini without any tubules after growing on-Matrigel for 10 days (**Fig. 7E**). Thus, antisense mediated knock-down of CEACAM1 effectively blocks tubule formation of hPrECs grown on- Matrigel. Antisense-treated hPrECs formed larger acini than untreated cells. Given that over-expression of CEACAM1 can inhibit PC3 growth [7], CEACAM1 antisense might affect acini size by affecting cell growth. When cells had been treated with antisense CEACAM20 16 mer for 10 days, CEACAM20 expression decreased by about 50% (**Fig. 7B**) whereas mRNA level decreased about 20% (**Fig. S4B**). When transferred to Matrigel after pretreatment with CEACAM20 antisense oligonucleotides, cells formed smaller acini compared with CEACAM1 antisense 16 mers or untreated controls (**Fig. 7F**). All acini failed to form tubules. Based on the observation that down-regulation of CEACAM1 vs CEACAM20 had differential effects on morphogenesis, CEACAM1 and CEACAM20 play different but coordinate roles in prostate morphogenesis.

## Discussion

The hPrECs used in this study were shown to be intermediate epithelial cells. Since these cells were able to differentiate into organoids that resemble embryonic prostates on-Matrigel, we conclude they possess an intrinsic ability to differentiate.

It has been proposed that prostate cancer originates from luminal epithelial cells and that prostate intraepithelial neoplasia (PIN) is the precursor to carcinoma [36]. The main pathological difference between PIN and carcinoma is that the basal cell layer is still present in PIN and basal cell markers are absent in prostate carcinoma. However, the origin of prostate cancer from basal cells was recently suggested by Witte and coworkers [28] who reported that basal cells isolated from primary benign human prostate tissue and transfected with AR and AKT/ERG were able to establish prostate tumors in NSG mice. Since their transduced luminal epithelial cells failed to form grafts with tubules, while basal cells did, they concluded that prostate carcinoma originates from basal cells. While those results are consistent with a role for basal cell differentiation into tubule-like structures, they required transfection with several genes to render the cells immortal and their work excluded the CK8/18^+^CK5/14^+^ intermediate cells. In contrast, our studies suggest that intermediate prostate epithelial cells without the addition of any immortalizing genes can differentiate into tubule-like structures, requiring only exposure to ECM.

The reproducible development of characteristic organoids on-Matrigel by hPrECs suggests that these cells possess an inherent program that spontaneously generates acini and tubules when exposed to ECM. Prostate development is a precise temporal and spatial process that begins at the 10th week of gestation with budding from the urogenital sinus (UGS) followed by elongation of solid cords of epithelial cells. By the 11th week, lumina form within the epithelial cords and the cellular end buds form primitive acini followed by extensive branching morphogenesis thereafter [26]. Using time-lapse microscopy we observed that hPrECs on-Matrigel formed elongated webs with cords, followed by spherical acini with hollow tubules. This sequence of events, including the striking morphological changes over time, in many respects resembles actual prostate development.

Comparing our results to other studies, Lang et al. [37] showed that freshly isolated prostate cells (a mixture of luminal and basal epithelial cell by their phenotype analysis) formed buds and ductal structures in-Matrigel but at a low incidence. In our on-Matrigel system, hPrECs formed >80% acini with tubules, while in-Matrigel tubules failed to form, suggesting that tubule formation was inhibited by direct contact to ECM. When RWPE-1 cells (non-tumorigenic prostate epithelium cell line immortalized with papilloma virus 18) were cultured in Matrigel, web-like structures formed, followed by formation of polarized acini without tubules [38]. These results correlate with our observation that hPrECs form web-like structures early followed by acini formation in-Matrigel.

Since hPrECs are clearly capable of undergoing a differentiation process, one can ask at least three questions. First, do the organoids resemble actual prostate morphogenesis, whether at a very early or late stage of differentiation? We can answer this question in the affirmative since the embryonic development of the prostate includes both acini and tubule formation in which tubules eventually link up to the urethra [26]. Since our system lacks other tissue clues such as the prostatic fat pad and nearby urethra, we can only say that the organoids represent an early and incomplete stage of differentiation. The second question is whether the molecular expression pattern resembles that found in the mature normal prostate. In our study, the expression of CEACAM20 is not only found in the lumen but also on the surface in contact with Matrigel. In this regard, Pearson et al. reported that RWPE1 cells formed acini with the central lumen in-Matrigel but PSA was only expressed on the cells in contact with Matrigel [39]. The reason for this is not clear but it is a matter of debate as to what constitutes the “luminal” surface in these assays. Third, is this particular “acinar-tubular” morphology donor dependent and/or isolation method dependent? When hPrECs purchased from either Lifeline Cell technology or Lonza and grown on- Matrigel, acini with tubules were formed (data not shown).

Since blocking CEACAM1 in breast epithelial cells in Matrigel cultures with antibodies or soluble CEACAM1 inhibited lumen formation [16], we asked if the same would be true for CEACAM1 and CEACAM20 for prostate epithelial cells. When CEACAM1 was blocked with antibodies or sCEACAM1 in hPrECs, lumina and tubule structures disappeared. Since CEACAM1 was expressed first during acinar development, we conclude that CEACAM1 plays perhaps two interrelated roles, lumen development of the acinus, and tubule sprouting. Since the tubule has a lumen continuous with the acinar lumen, it is likely that lumen development is a critical event required for tubule development. Thus, the antibody and sCEACAM1 blocking studies correlate with the differential organoid expression patterns of CEACAM1.

In addition, we performed knock-down studies using the novel gymnotic approach which requires micromolar amounts of antisense oligonucleotides in the medium for prolonged periods ([9], [33]). Although the knock-down efficiency appears less that what is usually observed with RNAi, about 50% in this case, there was a clear phenotype difference compared to hPrECS before and after knock-down of CEACAM1. In fact, the knock-down of tubule formation was identical to the antibody blocking study. Since RNAi required a transfection agent for delivery, resulting in massive cell death, it was impossible to compare antisense vs RNAi. On the other hand, since CEACAM20 antibodies had no effect on differentiation, we were unable to perform blocking experiments with antibodies for this marker. However, antisense gymnosis to CEACAM20 was successful and not only caused inhibition of tubule formation, but also significantly reduced the size of acini. Since CEACAM20 expression is confined to the acinus, these results suggest that normal acinus formation depends on CEACAM20, but not on CEACAM1 expression.

Although inhibition of CEACAM1 or CEACAM20 demonstrate that the two proteins are indispensable for establishment of normal luminal and tubular structures, the mechanism by which they form a lumen was not established. We speculate that both CEACAM1 and CEACAM20 are required for lumen formation, but in the case of prostate, their roles may be segregated, one to acinus, the other to tubule. In our studies on breast epithelial cell lumen formation, we showed that CEACAM1 induces apoptosis in the central cells of acini [4]. This signaling involved the intrinsic apoptotic pathway [4] along with induction of CAPN9 and cleavage of PKC-δ [13]. In contrast, studies on prostate lumen formation suggests that polarized fluid movement, not apoptosis, creates luminal space in the prostate, partially based on the observation that activated caspase 3 was expressed by very few cells and inhibition of polarized fluid movement prevented lumen formation [39]. Pathways implicated in prostate branching morphogenesis, include AR, Nkx3.1, Wnt, mTOR/PI3, BMP, FGF, and Hedgehog [27], [40]. Among these pathways CEACAM1 affects Wnt signaling in the small intestine [41]. CEACAM1 also associates with β-catenin while Ceacam1^−/−^ cells display increased glycogen synthase kinase 3-β (GSK3-β) phosphorylation as well as increased β-catenin nuclear expression [41]. Further studies on prostate cells are required to integrate these studies with CEACAM1. The clinical relevance of these studies suggests that both CEACAM1 and CEACAM20 can be used to further assess the degree of tumor differentiation.

In summary, our findings demonstrate an indispensable role for CEACAM1 and CEACAM20 in normal prostate luminal and ductal differentiation in a model system employing hPrECs and ECM. Since hPrECs can differentiate into organoids on ECM that resemble normal prostate development, the model system should be amenable to further studies aimed at differentiation and cancer. In this respect, the finding that loss of CEACAM1 and CEACAM20 is associated with loss of normal lumen structure in human high Gleason Grade prostate cancer is recapitulated in the in vitro model system.

## Supporting Information

Figure S1 **RT-PCR analysis of CEACAM20 expression in the various tissues.** From left to right: artery, bone marrow, brain, breast, duodenum, esophagus, heart muscle, small intestine, liver, lung, ovary, pancreas, pituitary, placenta, salivary gland, prostate, skeletal muscle, spleen, stomach, testis, thymus and uterus.
(TIF)

Figure S2 **Transmission electronic microcopy images of organoid formed by hPrECs at day 11 on Matrigel.** **A**. Base, **B**. Tubule, yellow arrow pointing at the vesicles in the lumen (L) and underneath the cell membrane of prostate epithelium cells (EP). Nanogold staining of CEACAM1 in tubule (**C**) and CEACAM20 in base (**D**), yellow arrow pointing at the positive nanogold particles.
(TIF)

Figure S3 **Inhibition of tubule formation by anti-CEACAM1 antibody or soluble CEACAM1.** **A**. Morphology of untreated hPrECs grown on 2D Matrigel (CTRL), isotype control antibody treated, anti-CEA antibody treated (as a further control), anti-CEACAM1 antibody treated at 10, 20 and 50 µg/mL, and sCEACAM1 treated at 0.2, 0.4 and 0.8 µM for 5 days, scale bar 100 µm. **B**. Quantification of organoids with tubules (200 colonies counted for each treatment at day 5).
(TIF)

Figure S4 **Quantification of CEACAM1 and CEACAM20 expression in hPrECs treated with antisense oligos to CEACAM1 or CEACAM20 for 10 days.** RT-PCR analysis of CEACAM1 (**A**) and CEACAM20 (**B**) with GAPDH control. **C**. Western blot analysis of CEACAM1 with β-actin control.
(TIF)

Movie S1 **Time lapse photography of tubule formation.** Twelve hours after hPrECs were attached to Matrigel, cells were transferred into an incubated stage equipped with an inverted fluorescent microscope and phase contrast images were taken every 30 min. for seven days.
(RAR)

Table S1 **Sequences of Oligonucleotides.** **A**: primers for PCR. **B**: primers for real time PCR. **C**: primers for CEACAM20 cloning. **D**: sequences of antisense oligonucleotides.
(DOCX)

Table S2 **Quantification of tubules in colonies.** **A**, at day 5 and **B**, at day 11. Antibody or soluble CEACAM1 were added at day2.
(DOCX)

## References

[1] Wagener C, Ergun S (2000) Angiogenic properties of the carcinoembryonic antigen-related cell adhesion molecule 1. Exp Cell Res 261: 19–24. 11082271 10.1006/excr.2000.5038

[2] Najjar SM (2002) Regulation of insulin action by CEACAM1. Trends Endocrinol Metab 13: 240–245. 12128284 10.1016/s1043-2760(02)00608-2

[3] Abou-Rjaily GA, Lee SJ, May D, Al-Share QY, Deangelis AM, et al (2004) CEACAM1 modulates epidermal growth factor receptor–mediated cell proliferation. J Clin Invest 114: 944–952. 15467833 10.1172/JCI21786 PMC518664

[4] Kirshner J, Chen CJ, Liu P, Huang J, Shively JE (2003) CEACAM1-4S, a cell-cell adhesion molecule, mediates apoptosis and reverts mammary carcinoma cells to a normal morphogenic phenotype in a 3D culture. Proc Natl Acad Sci U S A 100: 521–526. 12522268 10.1073/pnas.232711199 PMC141028

[5] Zebhauser R, Kammerer R, Eisenried A, McLellan A, Moore T, et al (2005) Identification of a novel group of evolutionarily conserved members within the rapidly diverging murine Cea family. Genomics 86: 566–580. 16139472 10.1016/j.ygeno.2005.07.008

[6] Busch C, Hanssen TA, Wagener C, O'Brink B (2002) Down-regulation of CEACAM1 in human prostate cancer: correlation with loss of cell polarity, increased proliferation rate, and Gleason grade 3 to 4 transition. Hum Pathol 33: 290–298. 11979369 10.1053/hupa.2002.32218

[7] Comegys MM, Carreiro MP, Brown JF, Mazzacua A, Flanagan DL, et al (1999) C-CAM1 expression: differential effects on morphology, differentiation state and suppression of human PC-3 prostate carcinoma cells. Oncogene 18: 3261–3276. 10359532 10.1038/sj.onc.1202666

[8] Tilki D, Irmak S, Oliveira-Ferrer L, Hauschild J, Miethe K, et al (2006) CEA-related cell adhesion molecule-1 is involved in angiogenic switch in prostate cancer. Oncogene 25: 4965–4974. 16568082 10.1038/sj.onc.1209514

[9] Stein CA, Hansen JB, Lai J, Wu S, Voskresenskiy A, et al (2010) Efficient gene silencing by delivery of locked nucleic acid antisense oligonucleotides, unassisted by transfection reagents. Nucleic Acids Res 38: e3. 19854938 10.1093/nar/gkp841 PMC2800216

[10] You YH, Hefta LJ, Yazaki PJ, Wu AM, Shively JE (1998) Expression, purification, and characterization of a two domain carcinoembryonic antigen minigene (N-A3) in pichia pastoris. The essential role of the N-domain. Anticancer Res 18: 3193–3201. 9858883

[11] Schumann D, Huang J, Clarke PE, Kirshner J, Tsai SW, et al (2004) Characterization of recombinant soluble carcinoembryonic antigen cell adhesion molecule 1. Biochem Biophys Res Commun 318: 227–233. 15110777 10.1016/j.bbrc.2004.04.024

[12] Zhang Z, Shively JE (2010) Generation of novel bone forming cells (monoosteophils) from the cathelicidin-derived peptide LL-37 treated monocytes. PLoS One 5: e13985. 21085494 10.1371/journal.pone.0013985 PMC2981577

[13] Chen CJ, Nguyen T, Shively JE (2010) Role of calpain-9 and PKC-delta in the apoptotic mechanism of lumen formation in CEACAM1 transfected breast epithelial cells. Exp Cell Res 316: 638–648. 19909740 10.1016/j.yexcr.2009.11.001 PMC2868383

[14] Chen CJ, Kirshner J, Sherman MA, Hu W, Nguyen T, et al (2007) Mutation analysis of the short cytoplasmic domain of the cell-cell adhesion molecule CEACAM1 identifies residues that orchestrate actin binding and lumen formation. J Biol Chem 282: 5749–5760. 17192268 10.1074/jbc.M610903200

[15] Gencheva M, Chen CJ, Nguyen T, Shively JE (2010) Regulation of CEACAM1 transcription in human breast epithelial cells. BMC Mol Biol 11: 79. 21050451 10.1186/1471-2199-11-79 PMC2991322

[16] Huang J, Hardy JD, Sun Y, Shively JE (1999) Essential role of biliary glycoprotein (CD66a) in morphogenesis of the human mammary epithelial cell line MCF10F. J Cell Sci 112 Pt 23: 4193–4205. 10564638 10.1242/jcs.112.23.4193

[17] Berthois Y, Katzenellenbogen JA, Katzenellenbogen BS (1986) Phenol red in tissue culture media is a weak estrogen: implications concerning the study of estrogen-responsive cells in culture. Proc Natl Acad Sci U S A 83: 2496–2500. 3458212 10.1073/pnas.83.8.2496 PMC323325

[18] Goldstein AS, Drake JM, Burnes DL, Finley DS, Zhang H, et al (2011) Purification and direct transformation of epithelial progenitor cells from primary human prostate. Nat Protoc 6: 656–667. 21527922 10.1038/nprot.2011.317 PMC3092477

[19] Kirshner J, Hardy J, Wilczynski S, Shively JE (2004) Cell-cell adhesion molecule CEACAM1 is expressed in normal breast and milk and associates with beta1 integrin in a 3D model of morphogenesis. J Mol Histol 35: 287–299. 15339048 10.1023/B:HIJO.0000032360.01976.81 PMC7087591

[20] Prakash TP, Bhat B (2007) 2′-Modified oligonucleotides for antisense therapeutics. Curr Top Med Chem 7: 641–649. 17430205 10.2174/156802607780487713

[21] Yokoyama S, Chen CJ, Nguyen T, Shively JE (2007) Role of CEACAM1 isoforms in an in vivo model of mammary morphogenesis: mutational analysis of the cytoplasmic domain of CEACAM1-4S reveals key residues involved in lumen formation. Oncogene 26: 7637–7646. 17546042 10.1038/sj.onc.1210577

[22] Collins AT, Berry PA, Hyde C, Stower MJ, Maitland NJ (2005) Prospective identification of tumorigenic prostate cancer stem cells. Cancer Res 65: 10946–10951. 16322242 10.1158/0008-5472.CAN-05-2018

[23] Gudjonsson T, Villadsen R, Nielsen HL, Ronnov-Jessen L, Bissell MJ, et al (2002) Isolation, immortalization, and characterization of a human breast epithelial cell line with stem cell properties. Genes Dev 16: 693–706. 11914275 10.1101/gad.952602 PMC155359

[24] Benton G, George J, Kleinman HK, Arnaoutova IP (2009) Advancing science and technology via 3D culture on basement membrane matrix. J Cell Physiol 221: 18–25. 19492404 10.1002/jcp.21832

[25] Garraway LA, Lin D, Signoretti S, Waltregny D, Dilks J, et al (2003) Intermediate basal cells of the prostate: in vitro and in vivo characterization. Prostate 55: 206–218. 12692787 10.1002/pros.10244

[26] Timms BG (2008) Prostate development: a historical perspective. Differentiation 76: 565–577. 18462432 10.1111/j.1432-0436.2008.00278.x

[27] Ghosh S, Lau H, Simons BW, Powell JD, Meyers DJ, et al (2011) PI3K/mTOR signaling regulates prostatic branching morphogenesis. Dev Biol 360: 329–342. 22015718 10.1016/j.ydbio.2011.09.027 PMC3225010

[28] Goldstein AS, Huang J, Guo C, Garraway IP, Witte ON (2010) Identification of a cell of origin for human prostate cancer. Science 329: 568–571. 20671189 10.1126/science.1189992 PMC2917982

[29] Tilki D, Singer BB, Shariat SF, Behrend A, Fernando M, et al (2010) CEACAM1: a novel urinary marker for bladder cancer detection. Eur Urol 57: 648–654. 19487071 10.1016/j.eururo.2009.05.040

[30] Lauke H, Kilic N, Bozorgzad R, Fernando M, Neshat-Vahid S, et al (2004) Expression of carcinoembryonic antigen-related cell adhesion molecule-1 (CEACAM1) in normal human Sertoli cells and its up-regulation in impaired spermatogenesis. Mol Hum Reprod 10: 247–252. 14985475 10.1093/molehr/gah020

[31] Klaile E, Vorontsova O, Sigmundsson K, Muller MM, Singer BB, et al (2009) The CEACAM1 N-terminal Ig domain mediates cis- and trans-binding and is essential for allosteric rearrangements of CEACAM1 microclusters. J Cell Biol 187: 553–567. 19948502 10.1083/jcb.200904149 PMC2779236

[32] Watt SM, Teixeira AM, Zhou GQ, Doyonnas R, Zhang Y, et al (2001) Homophilic adhesion of human CEACAM1 involves N-terminal domain interactions: structural analysis of the binding site. Blood 98: 1469–1479. 11520797 10.1182/blood.v98.5.1469

[33] Soifer HS, Koch T, Lai J, Hansen B, Hoeg A, et al (2012) Silencing of gene expression by gymnotic delivery of antisense oligonucleotides. Methods Mol Biol 815: 333–346. 22131003 10.1007/978-1-61779-424-7_25

[34] Hawley P, Gibson I (1992) The detection of oligodeoxynucleotide molecules following uptake into mammalian cells. Antisense Res Dev 2: 119–127. 1392535 10.1089/ard.1992.2.119

[35] Dellinger DJ, Yamada CM, Caruthers MH (2004) Oligodeoxyribonucleotide analogs functionalized with phosphonoacetate and thiophosphonoacetate diesters. Curr Protoc Nucleic Acid Chem Chapter 4: Unit 4 24. 10.1002/0471142700.nc0424s18 18428930

[36] Parsons JK, Gage WR, Nelson WG, De Marzo AM (2001) p63 protein expression is rare in prostate adenocarcinoma: implications for cancer diagnosis and carcinogenesis. Urology 58: 619–624. 11597556 10.1016/s0090-4295(01)01311-5

[37] Lang SH, Stark M, Collins A, Paul AB, Stower MJ, et al (2001) Experimental prostate epithelial morphogenesis in response to stroma and three-dimensional matrigel culture. Cell Growth Differ 12: 631–640. 11751458

[38] Bello D, Webber MM, Kleinman HK, Wartinger DD, Rhim JS (1997) Androgen responsive adult human prostatic epithelial cell lines immortalized by human papillomavirus 18. Carcinogenesis 18: 1215–1223. 9214605 10.1093/carcin/18.6.1215

[39] Pearson JF, Hughes S, Chambers K, Lang SH (2009) Polarized fluid movement and not cell death, creates luminal spaces in adult prostate epithelium. Cell Death Differ 16: 475–482. 19096393 10.1038/cdd.2008.181 PMC2857323

[40] Prins GS, Putz O (2008) Molecular signaling pathways that regulate prostate gland development. Differentiation 76: 641–659. 18462433 10.1111/j.1432-0436.2008.00277.x PMC2824174

[41] Leung N, Turbide C, Balachandra B, Marcus V, Beauchemin N (2008) Intestinal tumor progression is promoted by decreased apoptosis and dysregulated Wnt signaling in Ceacam1-/- mice. Oncogene 27: 4943–4953. 18454175 10.1038/onc.2008.136

