# Supplementary figures and images for: Role of CEACAM1 and CEACAM20 in an In Vitro Model of Prostate Morphogenesis
Source: PLoS One. 2013 Jan 24;8(1):e53359. doi: 10.1371/journal.pone.0053359 (PMC3554727; doi:10.1371/journal.pone.0053359)

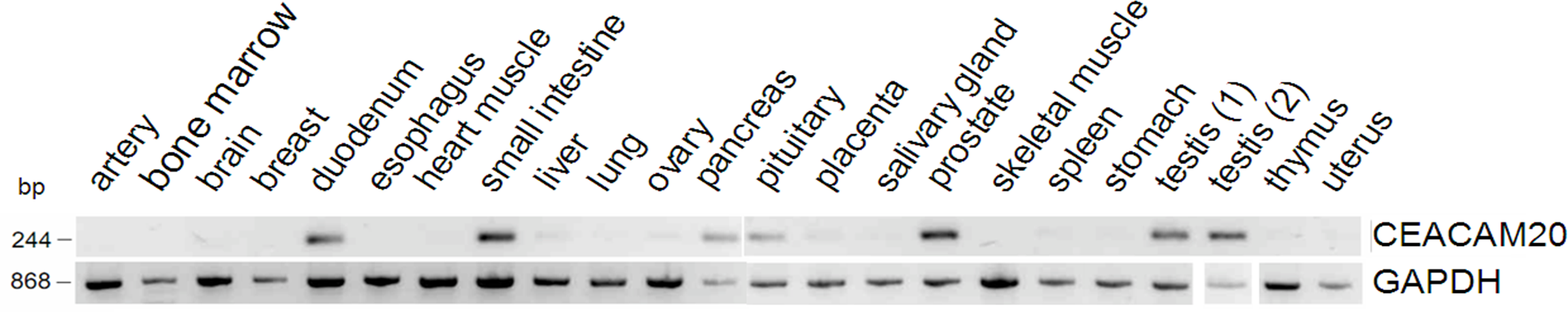

Supplement: Figure S1 — RT-PCR analysis of CEACAM20 expression in the various tissues. From left to right: artery, bone marrow, brain, breast, duodenum, esophagus, heart muscle, small intestine, liver, lung, ovary, pancreas, pituitary, placenta, salivary gland, prostate, skeletal muscle, spleen, stomach, testis, thymus and uterus. (TIF) [file pone.0053359.s001.tif]

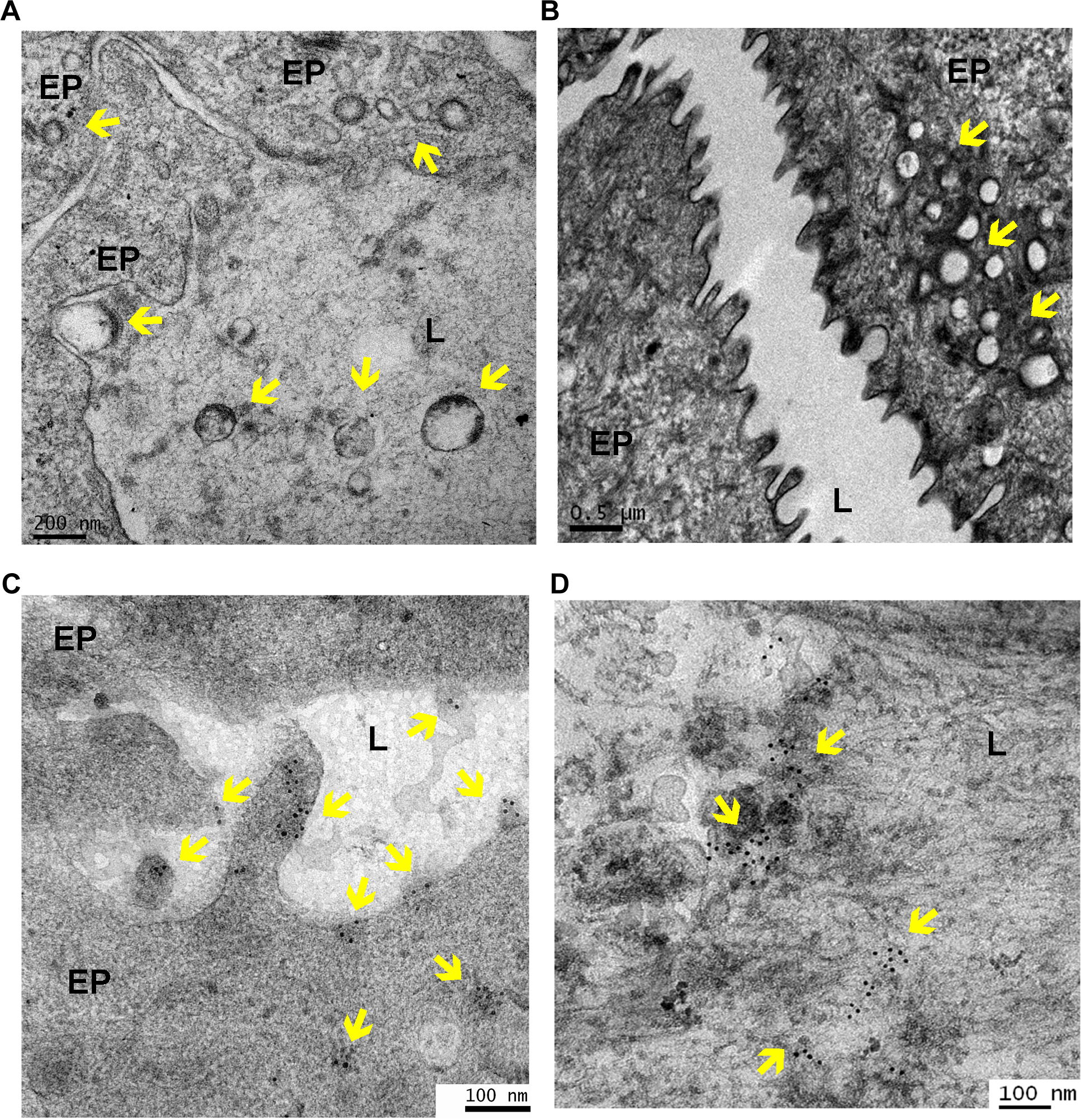

Supplement: Figure S2 — Transmission electronic microcopy images of organoid formed by hPrECs at day 11 on Matrigel. A. Base, B. Tubule, yellow arrow pointing at the vesicles in the lumen (L) and underneath the cell membrane of prostate epithelium cells (EP). Nanogold staining of CEACAM1 in tubule (C) and CEACAM20 in base (D), yellow arrow pointing at the positive nanogold particles. (TIF) [file pone.0053359.s002.tif]

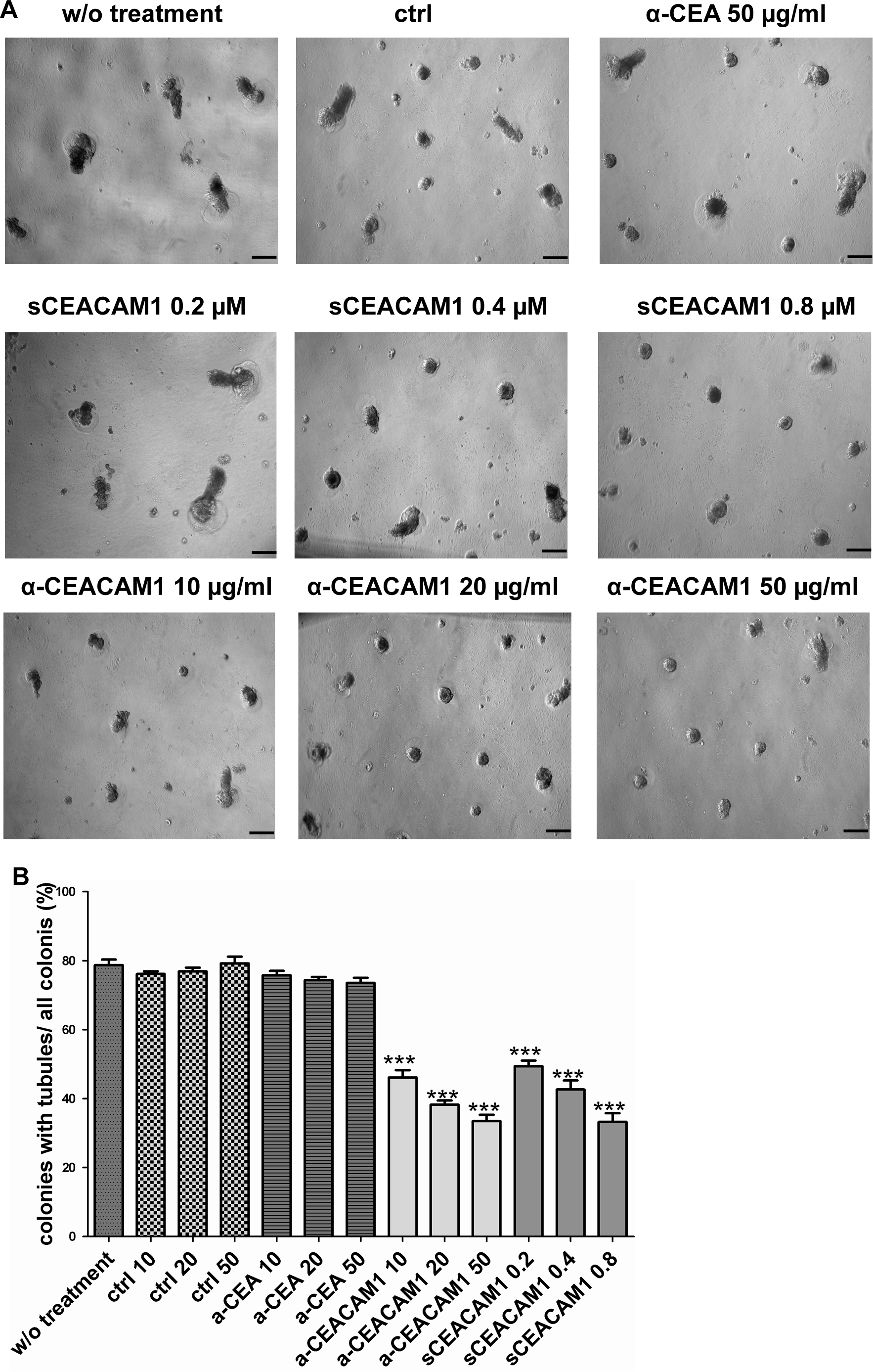

Supplement: Figure S3 — Inhibition of tubule formation by anti-CEACAM1 antibody or soluble CEACAM1. A. Morphology of untreated hPrECs grown on 2D Matrigel (CTRL), isotype control antibody treated, anti-CEA antibody treated (as a further control), anti-CEACAM1 antibody treated at 10, 20 and 50 µg/mL, and sCEACAM1 treated at 0.2, 0.4 and 0.8 µM for 5 days, scale bar 100 µm. B. Quantification of organoids with tubules (200 colonies counted for each treatment at day 5). (TIF) [file pone.0053359.s003.tif]

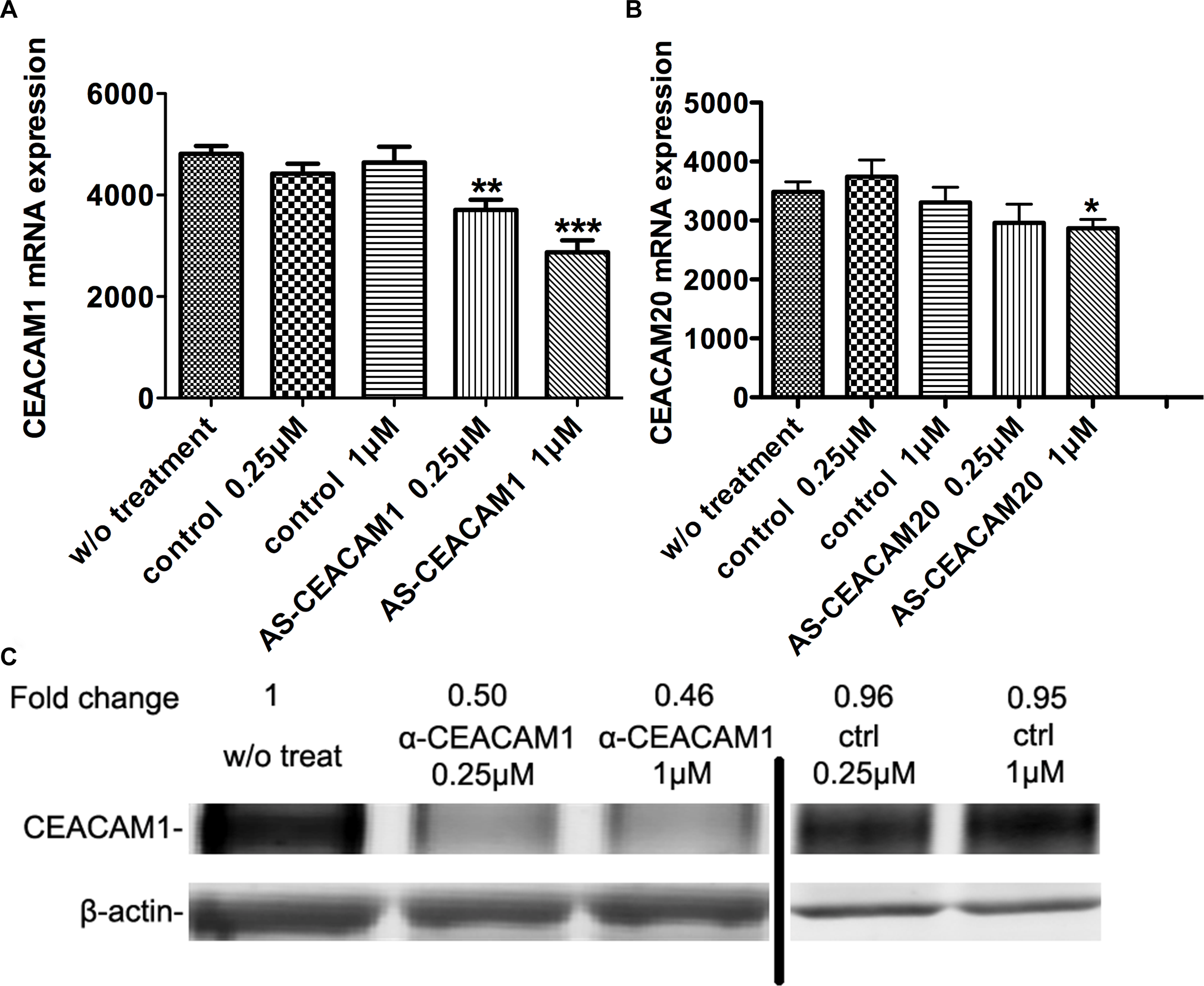

Supplement: Figure S4 — Quantification of CEACAM1 and CEACAM20 expression in hPrECs treated with antisense oligos to CEACAM1 or CEACAM20 for 10 days. RT-PCR analysis of CEACAM1 (A) and CEACAM20 (B) with GAPDH control. C. Western blot analysis of CEACAM1 with β-actin control. (TIF) [file pone.0053359.s004.tif]
