# Supplementary material for: Role of CEACAM1 and CEACAM20 in an In Vitro Model of Prostate Morphogenesis
Source: PLoS One. 2013 Jan 24;8(1):e53359. doi: 10.1371/journal.pone.0053359 (PMC3554727; doi:10.1371/journal.pone.0053359)
Supplement: Table S1 — Sequences of Oligonucleotides. A: primers for PCR. B: primers for real time PCR. C: primers for CEACAM20 cloning. D: sequences of antisense oligonucleotides. (DOCX) [file pone.0053359.s006.docx]

**Supplemental Table S1**

**A**

|  | Sequence | Amplicon Size (bp)^a^ | Ta (°C) |
| --- | --- | --- | --- |
| AR up | 5’-TACTTCGCCCCTGATCTGGTTTTC | 507 | 61 |
| AR down | 5’-CATGAGCTGGGGTGGGGAAATAGG |  |  |
| CD133 up | 5’-AGTGAGAAAGTGGCATCG | 400 | 55 |
| CD133 down | 5’-TCCTT GTAGACCCGAAAC |  |  |
| CEACAM1 up | 5’-AACGTCACCCAGAATGACA | 4L:1118;4S:1066;3L:831;3S:779; 1L:298; 1S:246 | 55 |
| CEACAM1 down | 5’-TCATTGGAGTGGTCCTGAG |  |  |
| CEACAM20 up | 5’-CAAAGTCTCACCCACCCTGT | 200 | 58 |
| CEACAM20 down | 5’-TGAGGCATGGTCAGTGTTTC |  |  |
| CK8 up | 5’-TGAGGTCAAGGCACAGTACG | 160 | 55 |
| CK8 down | 5’-TGATGTTCCGGTTCATCTCA |  |  |
| CK5 up | 5’-CGACAAGGTGCGGTTCCTG | 682 | 60 |
| CK5 down | 5’- GCAGATTGGCGCACTG |  |  |
| CK14 up | 5’-GATGACTTCCGCACCAAGTATGAG | 439 | 60 |
| CK14 down | 5’-TCAATCTCCAGGTTCTGCATGGTG |  |  |
| CK18 up | 5’-TGGTCACCACACAGTCTGCT | 347 | 50 |
| CK18 down | 5’-CCAAGGCATCACCAAGATTA |  |  |
| PAP up | 5’-GACCGAAGTCCCATTGACA | 771 | 55 |
| PAP down | 5’-AACATCTAGCGCCATCTGTAGG |  |  |
| PSA up | 5’-GGCAGGTGCTTGTAGCCTCTC | 521 | 61 |
| PSA down | 5’-CACCCGACGAGGTGCTTTTGC |  |  |
| PSCA up | 5’-TGCTTGCCCTGTTGATGGCAG | 321 | 60 |
| PSCA down | 5’-TGACCATGAAGGCTGTGCTGCTT |  |  |

^a^ bp: base pair; Ta: annealing temperature.

**B**

|  | Sequence | Amplicon Size (bp) |
| --- | --- | --- |
| CEACAM1 up | 5’-TGCTGGCATTGTGATTGGAG | 200 |
| CEACAM1 down | 5’-ACATGCCAGGGCTACTGCTATC |  |
| CEACAM20 up | N/A. Patent protection by Qiagen | 136 |
| CEACAM20 down | N/A. Patent protection by Qiagen |  |
| GAPDH up | 5’-CATTGCCCTCAACGACCACTTTGT | 73 |
| GAPDH down | 5’-CACCCTGTTGCTGTAGCCAAATTC |  |

bp: base pair; N/A: not available.

**C**

| CEA20-5UT-HindIII-f | GATCAAGCTTACGGCCTGGCACACTGAAG |
| --- | --- |
| CEA20UTR3-EcoRI-r | TATGAATTCATGGGAGCCAGGGCCTGAC |

**D**

|  | Sequence (16mer) |
| --- | --- |
| CEACAM1 antisense | 5’-ACAAUGCCAGCAAUGG |
| CEACAM20 antisense | 5’-GACCUAGCAUUCUCCA |
| scrambled control | 5’-AAAUGUACUGCGCGUG |
