# Supplementary material for: Role of CEACAM1 and CEACAM20 in an In Vitro Model of Prostate Morphogenesis
Source: PLoS One. 2013 Jan 24;8(1):e53359. doi: 10.1371/journal.pone.0053359 (PMC3554727; doi:10.1371/journal.pone.0053359)
Supplement: Table S2 — Quantification of tubules in colonies. A, at day 5 and B, at day 11. Antibody or soluble CEACAM1 were added at day2. (DOCX) [file pone.0053359.s007.docx]

**Supplemental Table S2**

**A**

| treatment | concentration | Colonies with tubules/ all colonies, counted at day 5 (%) |
| --- | --- | --- |
| w/o treatment^a^ | NA^a^ | 77.93 |
| α-CEA | 10 μg/mL | 77.07 |
| α-CEA | 50 μg/mL | 73.72 |
| sCEACAM1 | 0.2 μM | 67.61 |
| sCEACAM1 | 0.8 μM | 64.66 |
| α-CEACAM1 | 10 μg/mL | 65.04 |
| α-CEACAM1 | 50 μg/mL | 60.00 |

**^a^** w/o, without; NA, not applicable.

**B**

| treatment | concentration | Colonies with tubules/ all colonies, counted at day 11(%) |
| --- | --- | --- |
| w/o treatment | NA | 75.50 |
| α-CEA | 10 μg/mL | 75.26 |
| α-CEA | 50 μg/mL | 78.57 |
| sCEACAM1 | 0.2 μM | 71.43 |
| sCEACAM1 | 0.8 μM | 76.29 |
| α-CEACAM1 | 10 μg/mL | 73.68 |
| α-CEACAM1 | 50 μg/mL | 73.53 |
